# ECMELLA as a bridge to heart transplantation in refractory ventricular fibrillation: A case report

**DOI:** 10.3389/fcvm.2023.1074544

**Published:** 2023-02-13

**Authors:** Raphaël Giraud, Benjamin Assouline, Haran Burri, Dipen Shah, Philippe Meyer, Sophie Degrauwe, Matthias Kirsch, Karim Bendjelid

**Affiliations:** ^1^Intensive Care Unit, Geneva University Hospitals, Geneva, Switzerland; ^2^Faculty of Medicine, University of Geneva, Geneva, Switzerland; ^3^Geneva Hemodynamic Research Group, Geneva, Switzerland; ^4^Department of Cardiology, Geneva University Hospitals, Geneva, Switzerland; ^5^Cardiac Surgery, Cardiovascular Department, University Hospital and University of Lausanne, Lausanne, Switzerland

**Keywords:** VA-ECMO, Impella, ECMELLA, cardiac arrest, refractory ventricular fibrillation, heart transplantation

## Abstract

**Background:**

Extracorporeal membrane oxygenation (ECMO) is an effective cardiorespiratory support technique in refractory cardiac arrest (CA). In patients under veno-arterial ECMO, the use of an Impella device, a microaxial pump inserted percutaneously, is a valuable strategy through a left ventricular unloading approach. ECMELLA, a combination of ECMO with Impella, seems to be a promising method to support end-organ perfusion while unloading the left ventricle.

**Case summary:**

The present case report describes the clinical course of a patient with ischemic and dilated cardiomyopathy who presented with refractory ventricular fibrillation (VF) leading to CA in the late postmyocardial infarction (MI) period, and who was successfully treated with ECMO and IMPELLA as a bridge to heart transplantation.

**Discussion:**

In the case of CA on VF refractory to conventional resuscitation maneuvers, early extracorporeal cardiopulmonary resuscitation (ECPR) associated with an Impella seems to be the best strategy. It provides organ perfusion, left ventricular unloading, and ability for neurological evaluation and VF catheter ablation before allowing heart transplantation. It is the treatment of choice in cases of end-stage ischaemic cardiomyopathy and recurrent malignant arrhythmias.

## Introduction

Ventricular fibrillation (VF) is a life-threatening situation leading to cardiac arrest (CA) (1). Emergency treatment for VF includes cardiopulmonary resuscitation (CPR), antiarrhythmic drugs, and external electrical shocks (EES) with a defibrillator (2). Refractory ventricular fibrillation (RVF) is thought to be defined as failure to obtain return of spontaneous circulation (ROSC) within 10 min despite 3 defibrillation attempts, 3 mg of epinephrine, and 300 mg of amiodarone (3). The high mortality rates during VF [between 85 and 97% (4)] have brought interest in the development of a combined approach of conventional resuscitation techniques by external cardiac compressions and defibrillation with extracorporeal life support through the use of extracorporeal membrane oxygenation (ECMO) (5). Thus, extracorporeal cardiopulmonary resuscitation (ECPR) has become a lifesaving approach for patients suffering a CA that is deemed refractory to conventional resuscitation (6). Most of the time, reperfusion of the myocardium by ECMO makes it possible to obtain ROSC (7). In some rarer cases, VF persists despite electric shocks and injection of antiarrhythmic agents (8). ECMO allows for continued resuscitation and organ perfusion, including to the brain. However, it does not allow for the unloading of the left heart chambers, increasing the risk of subendocardial ischemia and interfering with myocardial recovery. Further, by increasing afterload, ECMO carries the risks of potential atrioventricular thrombosis and refractory pulmonary oedema. Several more or less invasive techniques, such as intra-aortic balloon pump (IABP), have been described to unload the left ventricle (LV), including Impella-CP, a microaxial pump inserted through the aortic valve into the left ventricle *via* the femoral and percutaneous routes. According to some recent studies, this device reduces the mortality of patients in cardiogenic shock placed under VA-ECMO (9–11). In this regard, ECMELLA, a combination of ECMO with Impella, seems to be promising to support end-organ perfusion without causing further damage to the heart (12).

We describe the clinical course of a patient with end-stage ischemic and dilated cardiomyopathy and refractory VF leading to CA in the late postmyocardial infarction (MI) period with persistent severe LV dysfunction, who was successfully treated with ECMO and IMPELLA as a bridge to heart transplantation (HT).

## Case description

A 52-year-old patient with a history of active smoking was hospitalized due to severe ischemic heart failure. A coronary angiogram performed September 2021 showed severe three-vessel coronary disease with no possibility of revascularization, confirmed by PET-CT and MRI showing transmural necrosis affecting more than 55% of the LV with no residual ischemia. Transthoracic echocardiography showed an LV ejection fraction of 20–25% and an apical thrombus measuring 6 mm × 7 mm. The patient presented with syncope caused by VF treated with an external electric shock delivered by a Lifevest^®^ on October 4, 2021, followed by implantation of an ICD the same day.

On October 14, 2021 at 18:00, the patient reported having felt his defibrillator administered several shocks. Nine new shocks were delivered by the patient’s ICD. At 18:40, the patient presented with CA secondary to refractory VF. CPR was started immediately. The patient received 17 internal shocks, then 8 EES at 200 joules, four doses of 1 mg of adrenaline IV, two doses of 150 mg amiodarone IV, two doses of 100 mg lidocaine IV, and 5 mg metoprolol IV. At 19:00, CPR was continued using a LUCAS 2™ device, orotracheal intubation was performed, and the ECMO alarm was triggered. At 19:30, VA-ECMO was implanted at the bedside under transesophageal echocardiography guidance (no-flow 0 min and low-flow 50 min). A new coronary angiography showed an unchanged coronary status. While in the cath-lab, 4 new EESs were delivered, and the patient was administered amiodarone 300 mg IV, lidocaine 100 mg IV, metoprolol 5 mg IV, and 2 g of magnesium IV.

**FIGURE 1:**
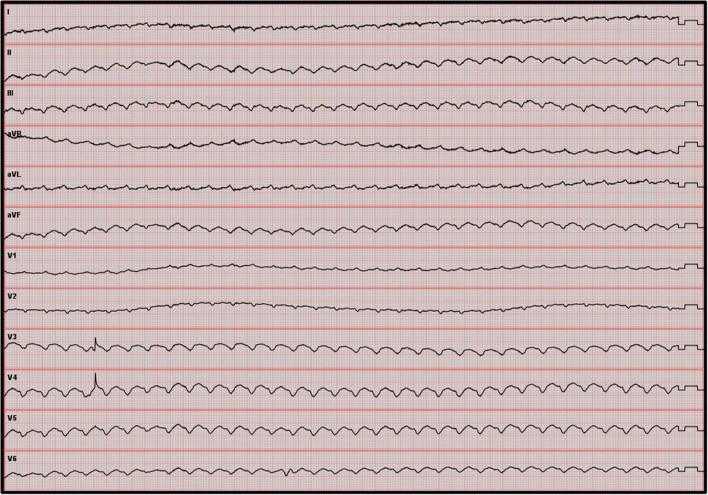
Twelve-lead ECG showing a ventricular flutter. ECG, electrocardiogram.

**FIGURE 2:**
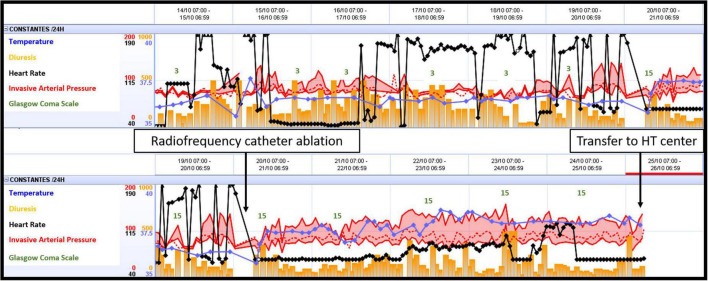
Time course of hemodynamic and neurologic parameters in the ICU while on ECMELLA. ECMELLA, combination of extracorporeal membrane oxygenation and Impella; HT, heart transplant.

Ventricular fibrillation remained refractory, and an LV discharge by Impella-CP was implanted. In the ICU, the patient was sedated and placed on amiodarone IV infusion at a rate of 1200 mg/day and a lidocaine IV infusion at a rate of 8 mg/h. VF turned into a persistent ventricular flutter (VFl) (Figure 1) despite multiple attempts of EES. Occasionally a sinus rhythm appeared spontaneously or with the waning of EES, however this persisted for only a few minutes. On VA-ECMO at 4.5 l/min and Impella between P2 and P4 generating a blood flow between 1 and 2 l/min, the patient was hemodynamically stabilized with low doses of norepinephrine. Lactate levels, renal failure, and liver function normalized within 48 h. In view of the uncertain neurological prognosis, electrophysiologists were reluctant to invasively treat this VF. On the sixth day, the sedation was discontinued. The patient woke up without neurological deficits. Thus, radiofrequency catheter ablation was performed with a satisfactory result. However, the patient presented with some recurrences of VF. The time course of hemodynamic and neurologic parameters in the ICU while on ECMELLA are shown in Figure 2. In view of end-stage heart failure and persistent rhythmic instability, this patient was registered for emergency HT and transferred to the HT center. This was performed on Day 29 without complications. The postoperative follow-ups were simple, and a transfer to rehabilitation occurred 4 weeks after HT.

## Comment

The present case report highlights that when the heart is no longer able to eject blood as in VF, the association between VA-ECMO and Impella allows to at the same time correctly perfuse the organs by solving the state of shock and organ dysfunction, as well as adequately unload the heart, avoiding the occurrence of pulmonary oedema and intracardiac thrombosis.

Cardiac arrest is a real health problem with a survival rate of 10–20% for patients undergoing conventional CPR (13). The contribution of ECMO in refractory CA (ECPR) has become a lifesaving approach (5). Indeed, ECPR helps to ensure organ perfusion while the primary cause of CA is treated. Moreover, in patients with sustained VF, ECPR allows the establishment of sufficient perfusion of the injured myocardium that usually leads to a higher chance of ROSC and organ recovery (7). Unfortunately, in the present case, this was not the result as VF persisted despite ECPR, the administration of multiple antiarrhythmic treatments, and finally defibrillations.

In ECPR patients whose heart has restarted, LV dysfunction occurs frequently. The increase in LV afterload induced by ECMO and the low cardiac contractility require the establishment of an LV unloading system (14). Impella has been shown to improve outcomes in ECMO-assisted refractory cardiogenic shock patients (15). Recent studies demonstrated the clinical benefits of LV unloading in cardiogenic shock in the setting of acute myocardial infarction. In the short term, LV unloading using mechanical circulatory support aims to reduce infarct size, limit ventricular remodeling, and prevent the development of the heart failure syndrome (16). In our patient, the persistence of VF led to a total absence of cardiac contraction and ejection, enhancing the risk of pulmonary oedema and LV thrombosis. Faced with a refractory arrhythmia, ECMELLA, by amending organ dysfunction without overloading the heart, allowed the patient to awaken. This is a “sine qua none” condition for both the continuation of resuscitation and discussion with the patient.

Indeed, the outcome of patients with refractory CA is directly related to neurological prognosis (17), which is conditioned by the adequate selection of patients to benefit from ECPR (18). The factors contributing to a favorable prognosis are: the shortest possible no-flow with an AC in front of a witness and a CPR performed immediately by a professional; a shockable initial rhythm; and low-flow state of <60 min until establishment of ECMO (5). This patient met these criteria. Once reassured by the good neurological state, the treatment of the refractory VF was carried out according to the recommendations by a radiofrequency ablation catheter (19). Unfortunately, due to end-stage ischemic heart disease and recurrences of arrhythmias despite the treatments undertaken, only heart transplantation was a viable option in the short, medium, and long term, as HT remains the best option for end-stage heart failure patients (20), and is associated with improved outcomes and a better cost–effectiveness profile. In this regard, ECMELLA permitted the discontinuation of sedation, the treatment of the VF by radiofrequency thermoablation and the consent of the patient to be transplanted.

## Conclusion

In the case of CA on VF refractory to conventional resuscitation maneuvers, early ECPR associated with an Impella seems to offer the best option to patients in terms of organ perfusion, left ventricular unloading, radiofrequency catheter ablation, and neurological evaluation. The present latter condition is a “sine qua none” condition that allows patients to be ethically consented for a heart transplant.

## Data availability statement

The raw data supporting the conclusions of this article will be made available by the authors, without undue reservation.

## Ethics statement

Ethical review and approval was not required for the study on human participants in accordance with the local legislation and institutional requirements. Written informed consent was obtained from the patient for publication of their health data.

## Author contributions

RG, BA, and KB oversaw the acquisition, analysis, interpretation of data, and drafted the manuscript. HB, DS, PM, SD, and MK revised the manuscript critically for important intellectual content. All authors provided approval for publication of the content and agreed to be accountable for all aspects of the work in ensuring that questions related to the accuracy or integrity of any part of the work were appropriately investigated and resolved.
